# Effects of Rhythmic Auditory Stimulation on Gait and Motor Function in Parkinson's Disease: A Systematic Review and Meta-Analysis of Clinical Randomized Controlled Studies

**DOI:** 10.3389/fneur.2022.818559

**Published:** 2022-04-15

**Authors:** Lei Wang, Jin-lin Peng, Jian-bin Ou-Yang, Li Gan, Shuai Zeng, Hong-Yan Wang, Guan-Chao Zuo, Ling Qiu

**Affiliations:** ^1^Department of Rehabilitation Medicine, Hunan Provincial People's Hospital, The First Affiliated Hospital of Hunan Normal University, Changsha, China; ^2^Tongji Medical College, Tongji Hospital, Huazhong University of Science and Technology, Wuhan, China; ^3^Department of Pain, Chengdu Integrated TCM and Western Medicine Hospital, Chengdu, China; ^4^Sichuan Rehabilitation Hospital Affiliated of Chengdu University of Traditional Chinese Medicine, Chengdu, China

**Keywords:** parkinsonism, rhythmic auditory stimulation, RAS, meta-analysis, Parkinson's

## Abstract

**Objective:**

This study aimed to summarize the effectiveness of rhythmic auditory stimulation (RAS) for the treatment of gait and motor function in Parkinson's disease (PD) through a systematic review and meta-analysis.

**Methods:**

All studies were retrieved from eight databases. The effects of RAS on PD were determined using the following indicators: gait parameters including step length, stride width, step cadence, velocity, stride length; motor function including 6 min walk test (6MWT) and timed up-and-go test (TUGT); the Unified Parkinson's Disease Rating Scale (UPDRS); and the Berg Balance Scale (BBS). The risk map of bias of the quality of the studies and the meta-analysis results of the indicators was prepared with RevMan 5.2 software.

**Results:**

Twenty-one studies were included in the systematic review, and 14 studies were included in the meta-analysis. In the meta-analysis, the results of gait parameters, namely, velocity, step length, and stride length, were statistically significant (*P* < 0.05), whereas the results of cadence and stride width were not statistically significant (*P* ≧ 0.05). The results of 6MWT and TUGT for motor function as well as UPDRS-II, UPDRS-III, and BBS were statistically significant (*P* < 0.05).

**Conclusions:**

RAS could improve gait parameters, walking function, balance function, and daily living activities of individuals with PD. The application of RAS in conventional rehabilitation approaches can enhance motor performance in PD. Future studies should use a large sample size and a rigorous design to obtain strong conclusions about the advantages of RAS for the treatment of gait and motor function in PD.

## Introduction

Parkinson's disease (PD) is the second most common neurodegenerative disease worldwide, after Alzheimer's disease (1, 2). The prevalence of PD increases more rapidly than other neurological disorders. A previous study showed that about 6.1 million people worldwide received diagnosis of PD in 2016, observing a more than doubled incidence to that observed in 1990 (3). A study estimated that in 2020 ~930,000 people will receive diagnosis of PD in the US alone (4). In developed countries, PD affects about 1% of the over-60 years old population and 4% of people over 80 years (2). Patients with PD lose the autonomy and rhythm of movement and exhibit various gait abnormalities, such as changes in gait frequency and speed, failure of gait initiation, and freezing of gait (5); as the disease progresses, it even affects the patient's quality of life (6). The main symptoms of PD include abnormal balance and gait, which can lead to falls and fall injuries (7–9). According to statistics, two-thirds of patients with PD will have a fall experience annually (10), and more than 50% of the patients often have a risk of falling (11), which seriously endangers their safety and quality of life.

Treatment of PD involves pharmacologic and nonpharmacologic approaches (6). Pharmacologic approaches are the main intervention used to improve the motor dysfunction of patients, such as dopamine agonists, Levo-dopa (6). The dopaminergic drugs maximize their effects at early stages of the disease, reducing the rigidity and increasing the movement speed and amplitude (6). Along the disease course, these benefits tend toward a reduction due to the occurrence of other, no dopaminergic drugs responsive motor symptoms (such as axial problems, including balance difficulties) and because of side effects (12, 13). In addition, symptoms can become more severe as the disease progresses, and at the same time, they can become less responsive to drugs (14), often this implies a gradual increases of dopaminergic drugs dosage, sometimes leading to disagreeable side effects, such as fluctuations, freezing, dyskinesias or non-motor side-effects (6, 13, 15). For the above reasons, in order to optimize use of medication and cope with the progression of the patient's symptoms, pharmacologic approaches is often accompanied by therapies.

Nowadays neurorehabilitation is considered aa a crucial complementary therapy in order to provide a good PD management since the early stages of the disease. Different rehabilitative methods have been applied and some of them reached good evidence of efficacy as testified by the Cochrane review (16, 17). In the context of physical therapy, both gait and balance training play a key-role in PD management. The motor-cognitive exercises exploit the attentional and volitional functions and are considered as the most effective in this field (18). When adopting cueing techniques patients are required to adapt their performance to specific signals, which could be of different nature: visual (19), vibrotactile (20), auditory (21), others (18). Rhythmic auditory stimulation (RAS) is an auditory-based cueing technique (22). One of the earliest and relevant studies about the use of RAS in PD rehabilitation was conducted by Thaut and colleagues in 1996: they observed that RAS is effective in improving gait parameters such as speed, rhythm, and stride length (23).

In the following 20 years, the treatment of PD with RAS has been widely investigated and these conclusions were confirmed (2, 6, 24–28). RAS can increase the swing phase and reduce the support phase in the gait cycle (29), improves balance, reduces the risk of falls (30, 31), positively affecting daily living activities (32). RAS has received extensive attention due to its non-invasive operation, safety, easy accessibility, and lack of adverse effects (33). However, a scientific basis for the effect of RAS on gait requires evidence from high-quality evidence-based medicine. To date, previous studies employed different and small sample size. A meta-analysis on six studies reported intervention that not only included RAS (33). Two meta-analyses (26, 34) included a large number of samples, but their inclusion criteria were not limited to clinical randomized controlled trials (RCTs).

In this study, a meta-analysis of clinical randomized controlled trials was conducted to evaluate the effect of RAS on the treatment of PD. This work aims to provide strong evidence for the use of RAS to treat PD and analyze the deficiencies of previous studies.

## Methods

This systematic review was planned and conducted according to the Preferred Reporting Items for Systematic Reviews and Meta-Analyses (PRISMA) Guideline and the Cochrane Collaboration (35).

### Study Strategy

All articles were retrieved from English databases (Pubmed, Cochrane Library, Ovid, and Web of Science) and Chinese databases (China National Knowledge Infrastructure, WanFang Data, and Technology Periodical Database). The preliminary search time was limited to December 31, 2020, and the final search for other potential articles was completed on February 10, 2021. The languages of the studies were Chinese and English. We performed several pre-searches based on Mesh terms and (or) free words and determined the final search formula. The search words were as follows: parkinsonism, Parkinson's disease, rhythmic auditory stimulation, rhythmic auditory cue, and acoustic stimulation. Two independent authors (Wang Lei and Peng Jin-lin) conducted a literature search in accordance with the search strategy. If the results of the two independent authors differ, the third author (Qiu Ling) will participate in the discussion and decide the final consensus.

### Eligibility Criteria

The Population, Intervention, Comparison, Outcomes, Study Design (PICOS) framework (14) was used to de the eligibility criteria of the articles to be included in the review. Participants diagnosed with PD were included in the study. Studies that compared patients with PD with healthy people and frozen gait with non-freezing gait were excluded. Studies that used RAS as intervention and has a well-defined protocol that included information on the specific training parameters (type, time, intensity, frequency, and duration) were included. For comparison, studies should include interventions in the control group (such as drugs, rehabilitation training, etc.). Outcomes (for meta-analysis). The parameter evaluated were as follows: gait parameters including step length, stride width, step cadence, velocity, stride length; motor function including 6 min walk test (6MWT) and timed up-and-go test (TUGT); Parkinson's functional impairment using Unified Parkinson's Disease Rating Scale (UPDRS); and balance function using Berg Balance Scale (BBS). Only RCTs were included in the review.

### Study Selection

Two authors (Wang Lei and Peng Jin-lin) independently reviewed the title and abstract sections of the retrieved articles. First, we eliminated duplicate articles by using “Medical Literature King V6” software. Second, we excluded inappropriate articles after reading the title and abstract following the eligibility criteria in the PICOS framework (14). Finally, we downloaded the potentially relevant articles for a more detailed full-text review. If the results of the two independent authors differ, the third author (Qiu Ling) will participate in the discussion and decide the final consensus.

### Data Extraction

We extracted the following data: general information including first author, year of publication, sample size, gender, age, treatment course and intervention measures; outcome indicators including gait parameters such as step length, stride width, step cadence, velocity, and stride length; and motor function indices including UPDRS, 6MWT, BBS, and TUGT. Two authors (Wang Lei and Peng Jin-lin) independently reviewed the data according to the search strategy. If the results of the two independent authors differ, the third author (Qiu Ling) will participate in the discussion and decide the final consensus. When an included article had no valid data, whether valid data can be obtained by contacting the author of the article. If data were still unavailable, then the article was not included in the meta-analysis but was included in the systematic review.

### Risk of Bias

We evaluated the quality of the included studies. Scores were compared in a consensus meeting by two independent authors (Wang Lei and Peng Jin-lin). If the results of the two independent authors differ, the third author (Qiu Ling) will participate in the discussion and decide the final consensus. The Cochrane risk of bias assessment tool outlined in chapter 8 of the Cochrane Hand-book for Systematic Reviews of Interventions (Version 5.1.0) was used to assess the risk of bias of the articles. Each article was assessed for selection bias, performance bias, detection bias, attrition bias, and reporting bias. Each domain was rated as high risk of bias, unclear of bias, or low risk of bias (36). A risk map of bias of the studies was prepared with RevMan 5.2 software.

### Statistical Analysis

Separate meta-analyses were conducted considering the heterogeneity of the interventions and measures of outcome indicators. Sub-group meta-analyses and sensitivity analyses were used to determine whether the characteristics of the interventions had any influence on the effects of RAS on PD. The Review Manager 5.2 software of Cochrane Collaboration was used in the meta-analysis. The outcome variables were continuous, so the mean difference (MD) was calculated, and the 95% CI of the statistical results was reported. *P*-value <0.05 indicated statistical significance for an overall effect (*Z*). Chi-square test was used to calculate the heterogeneity of the included articles. When heterogeneity was *P* > 0.1 and *I*^2^ <50%, a fixed-effect model was used; when heterogeneity was *I*^2^ > 50%, the causes of heterogeneity were analyzed by subgroup analysis or sensitivity analysis. When the results still had heterogeneity, the random-effect mode was used for summary analysis (36).

## Results

### Search Results

**Figure 1:**
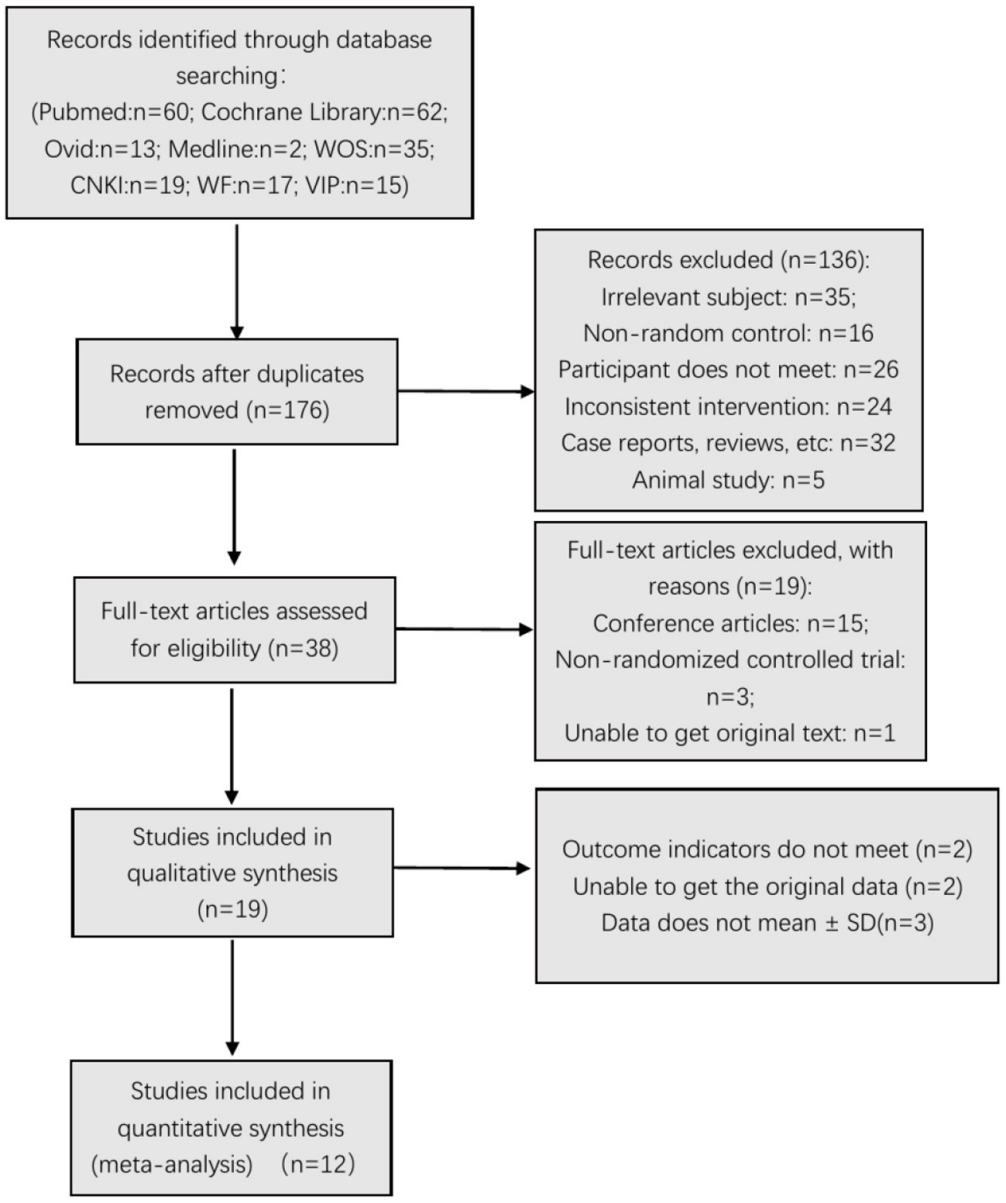
Flow chart of the search process.

In different stages of retrieval and screening, several articles were excluded. The detailed reasons and procedures are shown in Figure 1. A total of 223 abstracts were retrieved and imported into the Document Management Software of “Medical Literature King V6.” Among them, 47 duplicate studies were eliminated, and 136 articles were excluded after reading the title and abstract. Forty articles were left during the screening, and the full texts were downloaded for further screening. Fifteen articles were excluded because they were conference articles; three articles were excluded because they were non-randomized controlled trials; and one article was excluded because it did not contain original text. After deleting these articles, 19 were included in the qualitative analysis. After further reading the articles, two (37, 38) were excluded because their outcome indicators did not meet the inclusion criteria. Data of two articles (28, 39) only reported the *P*-value, and the original data could not be obtained even after contacting the article's author. Three articles (9, 40, 41) did not report the mean ± SD. Finally, 12 studies (6, 23, 29–32, 42–49) were included in the meta-analysis (six articles in Chinese and six articles in English).

### Risk of Bias

**Figure 2:**
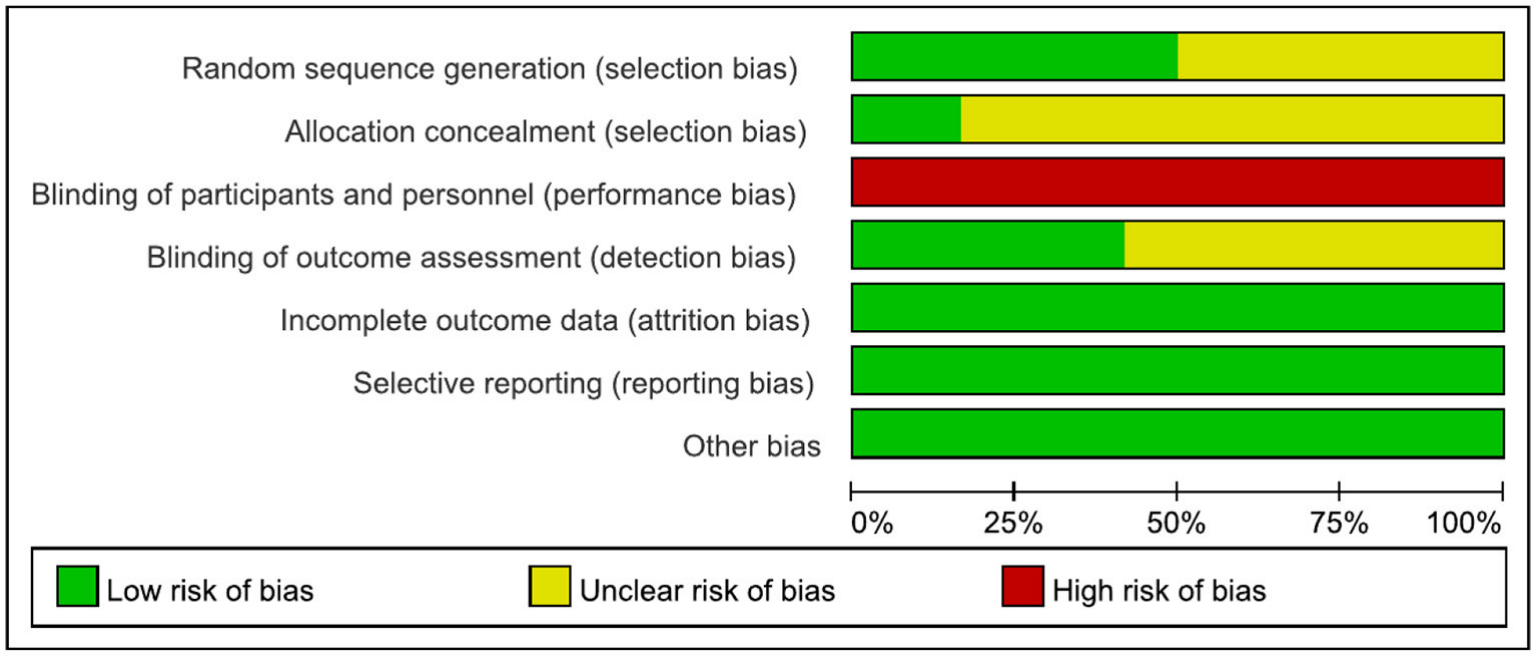
Risk of bias graph.

**Figure 3:**
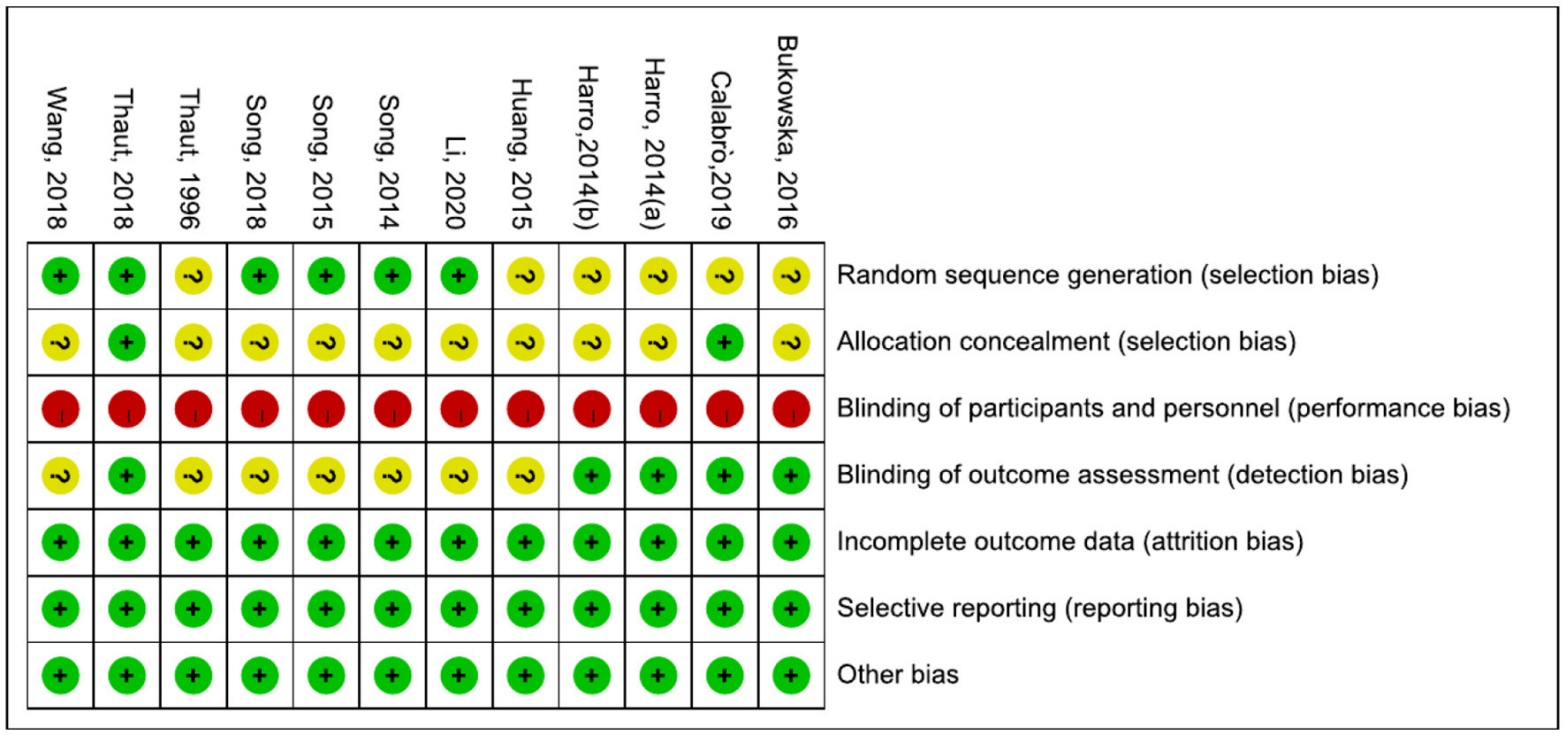
Risk of bias summary.

The results are shown in Figures 2, 3. The most common risk of bias of the studies was the lack of blinding of participants and trainers. Twelve studies were at high risk of bias for this reason. Blinding the participants and persons providing the treatment (trainers) is difficult. Six studies (6, 30, 32, 42, 43, 45–47) reported the source of random sequences, whereas the other studies did not specifically explain the random method used. Two studies (6, 30, 31) explained the implementation of allocation-hiding scheme, and the other studies did not specifically explain it. Five studies (6, 29–31, 48, 49) reported that the process of evaluating clinical outcomes was blinded, whereas the other studies did not describe whether the evaluation of the experimental results was blinded.

### Study Characteristics

Table 1 shows the information on participants, interventions, outcome indicators, diagnostic criteria, and follow up of the 12 studies included in the review. The sample sizes of the included studies were all small. The study with the largest sample size (44) had 90 participants, and those with the smallest sample size (48, 49) had 20 participants. The participants were mainly elderly, and the disease duration was uneven and considerably differed among the studies. Four studies (32, 42, 44, 46, 47) did not describe the Hoehn and Yahr scale of the participants, and seven studies (23, 29, 30, 44, 46, 48, 49) did not describe the MMSW of the participants. Six studies (6, 31, 32, 42, 43, 45–47) explained the diagnostic criteria of PD. Four studies (6, 31, 42, 43, 47) used the UK Brain Bank diagnostic criteria. One study (32, 45) diagnosed PD by using the Parkinson's diagnostic criteria of the European Movement Disorder Association. Another study (46) used the diagnostic criteria for PD in China (2016). Two studies (6, 48, 49) reported on follow up.

**Table 1:** Study characteristics.

**Author (design)**	**CON**	**EXE**	**Intervention CON**	**Intervention EXE**	**Outcome indicators**	**Diagnostic criteria**	**Follow up**
Song et al. (42)	*N* = 19 M/F = 12/9 A = 66.1 ± 7.9 DD(Y) = 6.7 ± 3.1 H&Y = NP MMSE = 28.4 ± 1.7	*N* = 20 M/F = 11/10 A = 65.7 ± 8.1 DD(Y) = 6.9 ± 2.9 H&Y = NP MMSE = 28.5 ± 1.9	Conventional anti-PD drug	Conventional anti-PD drug +RAS (10 minutes each time, three times a day for 3 weeks)	Step length, cadence, velocity, UPDRS-II, UPDRS-III, 6MWT, BBS	The UK Brain Bank diagnostic criteria	No
Song et al. (43)	*N* = 19 M/F = 10/10 A = 66.9 ± 7.9 DD(Y) = 6.7 ± 3.1 H&Y = 2.4 ± 0.6 MMSE = 28.5 ± 1.4	*N* = 18 M/F = 9/11 A = 65.7 ± 6.2 DD(Y) = 6.5 ± 3.3 H&Y = 2.4 ± 0.5 MMSE = 28.1 ± 1.3	Conventional anti-PD drug + weight-losing treadmill (30 min/time, 1 time/d, 5 d/week, 4 weeks)	Conventional anti-PD drug + treadmill training with RAS and visual stimulation (30 min/time, 1 time/d, 5 d/week, 4 weeks)	Step length, cadence, velocity, UPDRS-II, UPDRS-III, 6MWT, BBS	The UK Brain Bank diagnostic criteria	No
Huang et al. (44)	*N* = 45 M/F = 23/22 A = 68.7 ± 4.1 DD(Y) = 6.9 ± 3.0 H&Y = NP MMSE = NP	*N* = 45 M/F = 24/21 A = 65.2 ± 8.4 DD(Y) = 6.8 ± 2.8 H&Y = NP MMSE = NP	Conventional anti-PD drug	Conventional anti-PD drug + RAS and visual stimulation (30 min/time, 1 time/d, 5 d/week, 4 weeks)	Step length, cadence, velocity, UPDRS-2, UPDRS-3, 6MWT, BBS	NP	No
Song et al. (45)	*N* = 34 M/F = 18/16 A = 66.9 ± 7.9 DD(Y) = 6.7 ± 3.6 H&Y = 2.4 ± 0.5 MMSE = 27.8 ± 1.4	*N* = 34 M/F = 17/17 A = 66.5 ± 7.4 DD(Y) = 6.5 ± 3.3 H&Y = 2.4 ± 0.7 MMSE = 27.1 ± 1.7	Conventional anti-PD drug + weight-losing treadmill (30 min/time, 1 time/d, 5 d/week, 4 weeks)	Conventional anti-PD drug + treadmill training with RAS and visual stimulation (30 min/time, 1 time/d, 5 d/week, 4 weeks)	Step length, cadence, velocity, TUGT, BBS	Parkinson's diagnostic criteria of the European Movement Disorder Association	No
Li et al. (46)	*N* = 40 M/F = 24/16 A = 68.72 ± 3.26 DD(Y) = 3.52 ± 1.23 H&Y = NP MMSE = NP	*N* = 40 M/F = 27/13 A = 70.21 ± 3.24 DD(Y) = 3.87 ± 1.67 H&Y = NP MMSE = NP	Routine rehabilitation exercise training. 30 min/time, 2 times/day, continuous training for 1 month.	RAS. 10minutes/time, 4 times/day, and train continuously for 1 month.	BBS	Diagnostic criteria for Parkinson's disease in China (2016)	No
Wang et al. (47)	*N* = 43 M/F = 25/18 A = 71.42 ± 3.05 DD(Y) = 4.67 ± 1.13 H&Y = NP MMSE = 28.34 ± 1.69	*N* = 43 M/F = 23/20 A = 71.52 ± 2.98 DD(Y) = 4.33 ± 1.02 H&Y = NP MMSE = 28.53 ± 1.85	Routine rehabilitation training, 30min/time, 2 times/day, the number of trainings per week is maintained at 5 or 6 times, continuous training for 1 month.	C+ RAS, 10 min/time, 4 times/day, continuous training for 1 month.	Step length, cadence, velocity, UPDRS-III; 6MWT; BBS	the UK Brain Bank diagnostic criteria	No
Calabrò et al. (31)	*N* = 25 M/F = 6/14 A = 73 ± 8 DD(Y) = 9.3 ± 3 H&Y = 3 ± 1 MMSE = 25 ± 3	*N* = 25 M/F = 9/11 A = 70 ± 8 DD(Y) = 10 ± 3 H&Y = 3 ± 1 MMSE = 26 ± 3	30 min of non_RAS treadmill one time of day, 5 times per week for 8 weeks	30 min of_RAS treadmill one time of day, 5 times per week for 8 weeks	Cadence, velocity, stride length, BBS, TUGT	The UK Brain Bank diagnostic criteria	Yes
Harro et al. (48)	*N* = 10 M/F = 5/5 A = (45-75) DD(Y) = (1-6.5) H&Y = (1-3) MMSE = NP	*N* = 10 M/F = 8/2 A = (46-85) DD(Y) = (1-7) H&Y = (1-3) MMSE = NP	Speed-dependent treadmill training. 3 sessions of 30 min per week for 6 weeks	RAS 3 sessions of 30 min per week for 6 weeks	6MWT	NP	Yes
Thaut et al. (23)	*N* = 11 M/F = 8/3 A = 74 ± 3 DD(Y) = 5.4 ± 3 H&Y = 2.5 MMSE = NP	*N* = 15 M/F = 10/5 A = 69 ± 8 DD(Y) = 7.2 ± 4 H&Y = 2.4 MMSE = NP	Program (walking on a flat surface, stair stepping, and stop-and-go exercises) daily for 30 min, 3 weeks.	The program with RAS. daily for 30 min, 3 weeks.	Cadence, velocity, stride length	NP	No
Thaut et al. (30)	*N* = 22 M/F = 15/16 A = 73 ± 8 DD(Y) = 11.2 ± 6 H&Y = 3.4 MMSE = NP	*N* = 25 M/F = 17/13 A = 71 ± 7 DD(Y) = 10.9 ± 5 H&Y = 3.6 MMSE = NP	Trained daily with RAS daily for 30 min, 24 weeks (but discontinued training with RAS between weeks 8 and 16)	Trained daily with RAS daily for 30 min, 24 weeks	Cadence, velocity, stride length, TUGT, BBS,	NP	No
Harro et al. (49)	*N* = 10 M/F = 5/5 A = (45-75) DD(Y) = (1-6.5) H&Y = (1-3) MMSE = NP	*N* = 10 M/F = 8/2 A = (46-85) DD(Y) = (1-7) H&Y = (1-3) MMSE = NP	Speed-dependent treadmill training, 3 sessions of 30 min per week for 6 weeks	RAS, 3 sessions of 30 min per week for 6 weeks	BBS	NP	Yes
Bukowska et al. (29)	*N* = 25 M/F = 10/15 A = 63.44 ± 9.67 DD(Y) = 6.76 ± 4.32 H&Y = (2-3) MMSE = NP	*N* = 30 M/F = 15/15 A = 63.4 ± 10.61 DD(Y) = 5.5 ± 3.9 H&Y = (2-3) MMSE = NP	maintain daily life activities (changing of position, walking, walking stairs)	NMT program [(RAS, Patterned Sensory Enhancement (PSE), Therapeutic Instrumental Music Performance (TIMP)], 45 min of sessions, four times a week, 4 weeks.	Step length, stride width, cadence, velocity, stride length	NP	No

*A, age; CON, control group; EXP, experiment group; DD(Y), Disease Duration(Year); H&Y, Hoehn and Yahr scale; MMSE, Mini-Mental State Examination; M/F, male/female; N, number of participants; NP, not provided; UPDRS, Unified Parkinson's Disease Rating Scale;6MWD, 6 Minute Walk Test, 10MWD, 10-m Walk Test, BBS, Berg Balance Scale, TUGT, timed up-and-go test*.

## Outcome Analysis

### Gait Parameters

**Figure 4:**
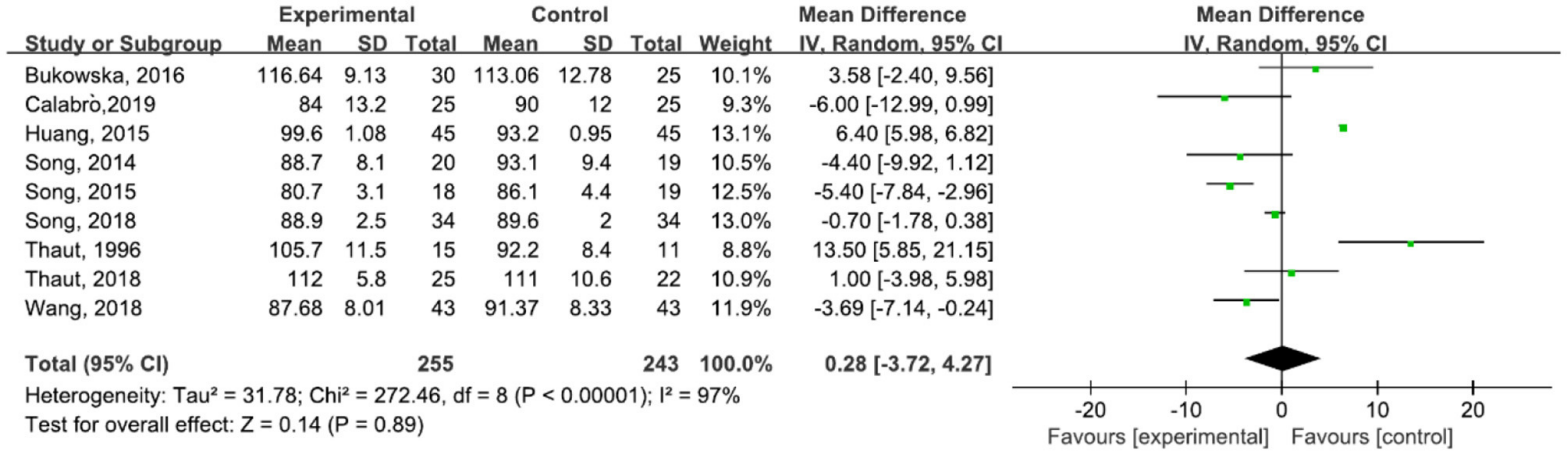
Forest plot showing MD (with 95% CI) for step cadence of the included studies comparing the experimental and control groups.

A total of 498 participants were included in nine studies on step cadence. The results showed heterogeneity (*I*^2^ = 97%). The result of the subgroup and sensitivity analyses showed no significant change in heterogeneity. We selected the random-effect model [MD = 0.28, 95% CI (−3.72, 4.27), *P* = 0.89]. The difference between the two groups was not statistically significant (Figure 4). Five studies (31, 42, 43, 45, 47) reported an increase in cadence after the treatment, and four studies (6, 23, 29, 30, 44) described a decrease in cadence after treatment.

**Figure 5:**
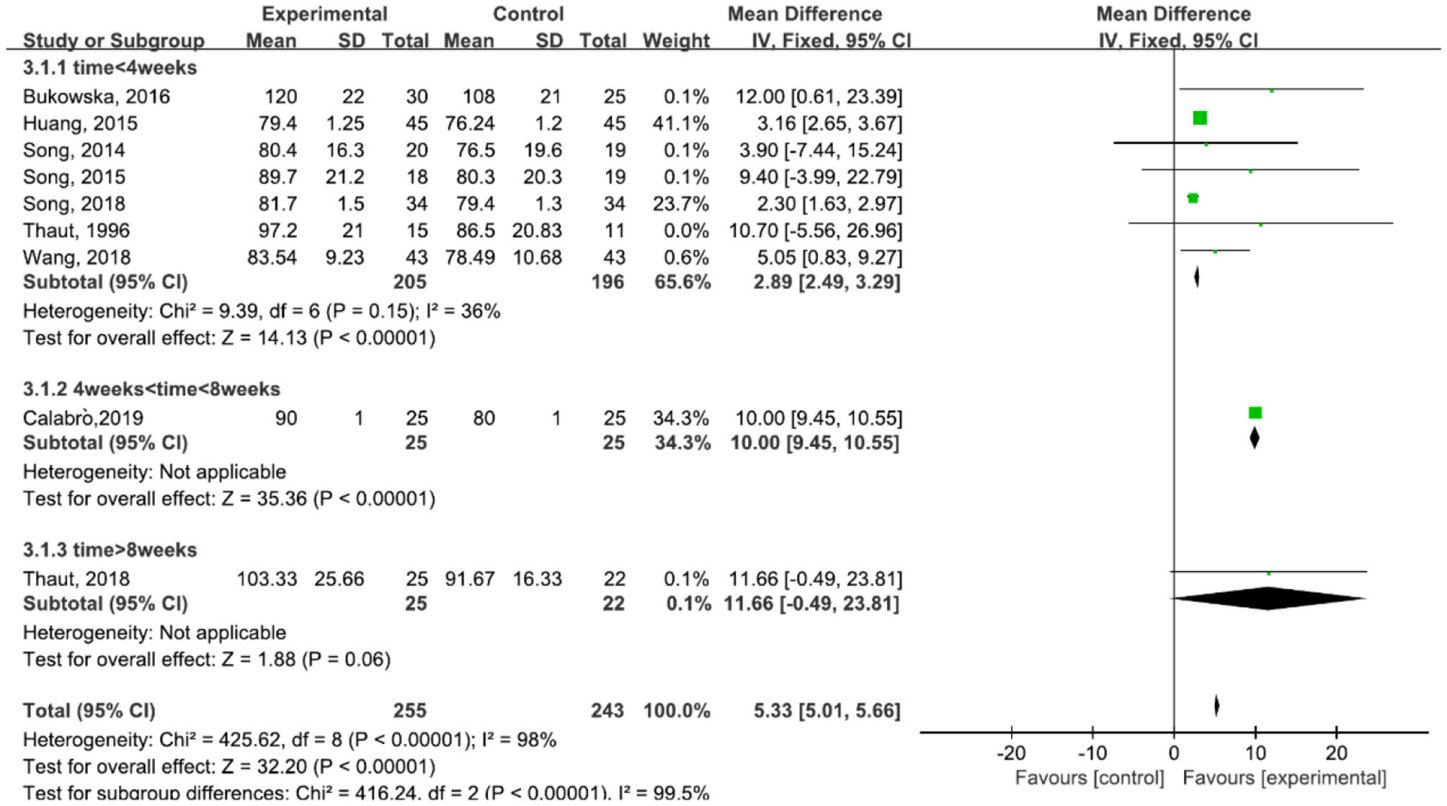
Forest plot showing MD (with 95% CI) for velocity of the included studies comparing the experimental and control groups.

A total of 498 participants were included in nine studies on velocity. The results showed heterogeneity (*I*^2^ = 98%). We performed subgroup analysis as follows: according to the time of treatment, the studies were divided into three subgroups: time ≦ 4 weeks, 4 weeks < time ≦ 8 weeks, and time > 8 weeks. The subgroup of time ≦ 4 weeks was included in seven studies (23, 29, 42–45, 47). The results showed *P* = 0.15 and *I*^2^ = 36%, so we selected the fixed-effect model [MD = 2.89, 95% CI (2.49, 3.29), (*P* < 0.00001)]. The subgroup of 4 weeks < time ≦ 8 weeks was included in one study (6, 31). The subgroup of time > 8 weeks was included in one study also (30). The results of the subgroup analysis was statistically significant (Figure 5).

**Figure 6:**
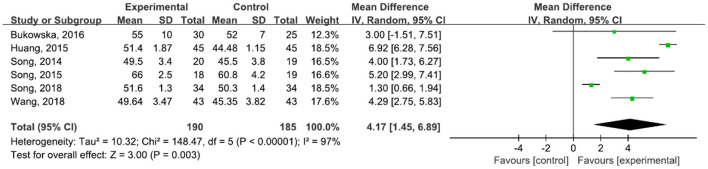
Forest plot showing MD (with 95% CI) for step length of the included studies comparing the experimental and control groups.

A total of 375 participants were included in six studies (6, 29, 42–45, 47) on step length. The results showed heterogeneity (*I*^2^ = 97%). The subgroup and sensitivity analyses showed no significant change in heterogeneity. We selected the random-effect model (MD = 4.17, 95% CI (1.45, 6.89), *P* = 0.003). The difference between the two groups was statistically significant (Figure 6).

**Figure 7:**
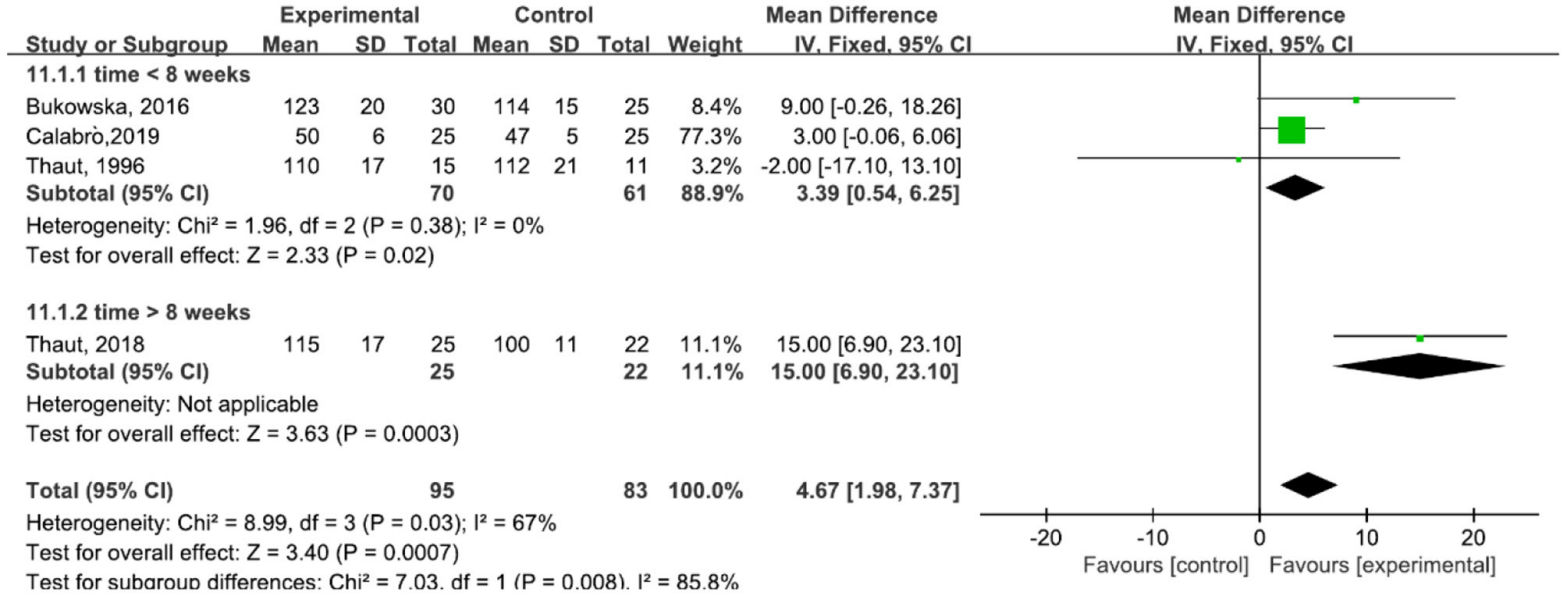
Forest plot showing MD (with 95% CI) for stride length of the included studies comparing the experimental and control groups.

A total of 178 participants were included in four studies (6, 23, 29–31) on stride length. The results showed heterogeneity (*I*^2^ = 67%). The subgroup and sensitivity analyses showed that the time of treatment was 16 weeks (30). This factor was analyzed as a possible cause of heterogeneity and was removed before performing another analysis. The results showed *P* = 0.58 and *I*^2^ = 0%, so we used a fixed-effect model [MD = 3.39, 95% CI (0.54, 6.25), *P* = 0.02]. The difference between the two groups was statistically significant (Figure 7).

### Motor Function

Walking function was described using three aspects: 6MWT, 10 m walk test (10MWT), and TUGT.

**Figure 8:**
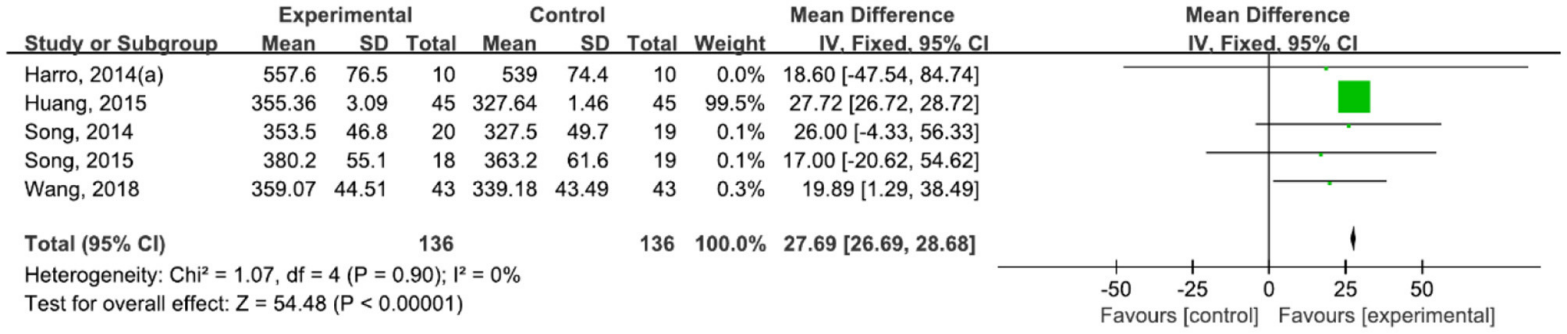
Forest plot showing MD (with 95% CI) for 6MWT of the included studies comparing the experimental and control groups.

A total of 272 participants were included in five studies (42–44, 47, 48) that used 6MWT. The results showed *P* = 0.90 and *I*^2^ = 0%, so we used a fixed-effect model [MD = 27.69, 95% CI (26.69, 28.68), (*P* < 0.00001)]. The difference between the two groups was statistically significant (Figure 8).

**Figure 9:**
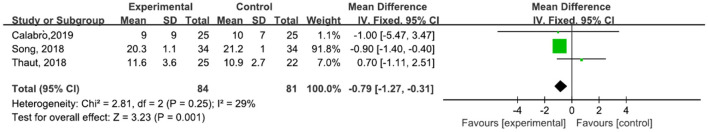
Forest plot showing MD (with 95% CI) for TUGT of the included studies comparing the experimental and control groups.

A total of 165 participants were included in three studies (30, 31, 45) that used TUGT. The results showed *P* = 0.25 and *I*^2^ = 29%, so we used a fixed-effect model [MD = −0.79, 95% CI (−1.27, −0.31) (*P* = 0.001)]. The difference between the two groups was statistically significant (Figure 9).

### UPDRS

In the study, UPDRS was described using two aspects: UPDRS-II and UPDRS-III.

**Figure 10:**
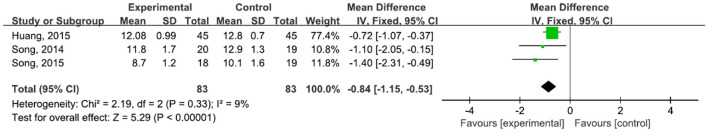
Forest plot showing MD (with 95% CI) for PDRS-II of the included studies comparing the experimental and control groups.

A total of 166 participants were included in three studies (42–44, 47) that used UPDRS-II. The results showed *P* = 0.33 and *I*^2^ = 9%, so we used a fixed-effect model [MD = −0.84, 95% CI (−1.15, −0.53), *P* < 0.0001)]. The difference between the two groups was statistically significant (Figure 10).

**Figure 11:**
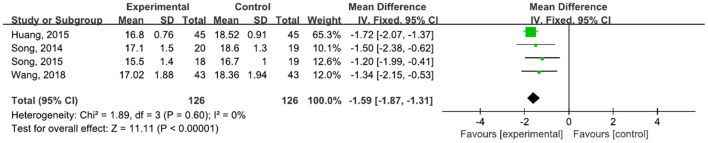
Forest plot showing MD (with 95% CI) for UPDRS-III of the included studies comparing the experimental and control groups.

A total of 252 participants were included in four studies (32, 42–44, 47) that used UPDRS-III. The results showed *P* = 0.60 and *I*^2^ = 0%, so we used a fixed-effect model [MD = −1.59, 95% CI (−1.87, −1.31), *P* < 0.0001]. The difference between the two groups was statistically significant (Figure 11).

### BBS

**Figure 12:**
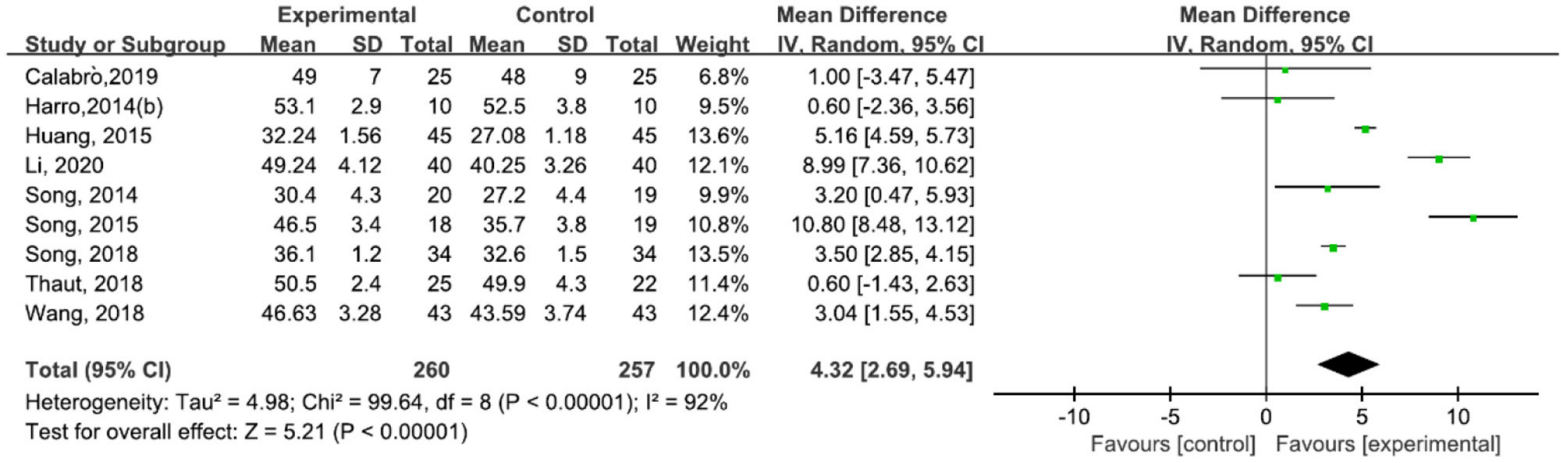
Forest plot showing MD (with 95% CI) for BBS of the included studies comparing the experimental and control groups.

A total of 517 participants were included in nine studies (30, 31, 42–47, 49) on BBS. The results showed heterogeneity (*I*^2^ = 92%). The subgroup and sensitivity analyses showed no significant change in heterogeneity. We selected the random-effect model [MD = 4.32, 95% CI (2.69, 5.94), *P* < 0.0001]. The difference between the two groups was statistically significant (Figure 12).

## Discussion

On the basis of the systematic review and meta-analysis results of this study, RAS could improve the gait quality, enhance motor and balance functions. In general, patients with PD are characterized with reduced speed and step length, increased step cadence and proportion of supporting phases, which could result in a frozen gait in the late stage of the disease (50). Based on the systematic review and meta-analysis results of the present study, RAS could improve the gait quality and enhance the motor and balance functions of patients with PD. The effectiveness of RAS for the treatment of PD has been extensively discussed (24–27, 34, 51–53).

As generated in Dorsal Striatum and because of depending on striatal dopaminergic tone, the Internal Pacing (IP) is altered in parkinsonian patients (18, 27, 54). In physiological movement conditions, the basal ganglia and the SMA establish a functional loop regulated by the IP, which represents a sort of Internal Cue System. In this concern, continuous pacing-induced adjustments play an important role in maintaining both movement rhythmicity and synchronization. With practice, this process becomes gradually automatized. This physiological path is lost in PD (27, 55, 56). Nevertheless, other networks affect movement rhythmicity and synchronization, mainly the External Entrainment (EE—based on an External Cueing System), which basically modulates motor behaviors with respect to environmental needs. As EE is the expression of cerebellar and prefrontal networks, it works synergically, as well as parallelly, with IP to express the best motor behavior in any environmental context. As it does not directly involve dopaminergic pathways, EE is spared in Parkinsonian patients for a long time along the disease course. This is the reason why it provides an effective compensation to restore the lost rhythmicity and synchronization of motor behavior in PD (27, 57, 58). RAS is a very good method to activate the EE and to optimize its synchronization with the IP, allowing to restore it (27, 59–62).

The result of the meta-analysis showed no statistical difference in step cadence. Some studies (31, 42, 43, 45, 47) have shown that RAS can reduce the step cadence of patients with PD, but the other studies (6, 23, 29, 30, 44) showed opposite results. Differences existed among the studies, but the results of each study showed that the RAS group is better than the control group in terms of change in step cadence. On the one hand, the reason may be the difference in the different stages of patients with PD. A previous study (24) pointed out that stride frequency is slightly reduced in the early stage of PD compared with that in the normal range. As the disease progresses, the patient's stride length decreases, and the stride frequency increases in a flustered gait. On the other hand, the reason for the analysis may be related to the step cadence of RAS because all studies did not specify a fixed and unified cadence of RAS. One study (29) did not specify the rhythm parameters of RAS, and another study (42) was based on the patient's gait frequency before intervention. Other studies added parameters based on the patient's walking frequency (44, 49). The cadence of RAS can be determined by computing the average walking speed of the patient according to the daily walking speed and developing a beat that matches the average walking speed of the patient through the software beat (39). Other studies used the rhythm of music to provide RAS training to the patients (29, 48). These reasons caused a large heterogeneity, so further research on the type and frequency of RAS intervention as well as the selection of the applicable frequency in the gait phase is needed.

In the meta-analysis, the heterogeneity of cadence, step length, and BBS was very high, and neither subgroup analysis nor sensitivity analysis could reduce the heterogeneity. The sample sizes of the included studies were too different, and the sample sizes were all small. In the included studies, the largest sample size was 90 participants (44), and the smallest sample size was only 20 participants (48, 49). A small sample size is prone to false-positive results, so the sample size must be increased. Therefore, the sample size must be increased in further work to improve the research quality.

## Future Perspective

However, whether RAS can be extended to home rehabilitation and daily life and whether this effectiveness in clinicals can be applied to home rehabilitation remain unclear. Considering the nature and economic burden of long-term Parkinson's, home rehabilitation is the first choice. Two included studies (32, 47) reported BI, but they only discussed the improvement of patients' ADL and did not mention the patients' application of RAS in ADL (like walking or climbing stair). The said studies had no safety report of patients' ADL. In a systematic review (11), 60.5% (range 35–90%) of the participants reported at least one fall, and 39% (range 18–65%) reported recurrent falls. Whether patients with PD will fall due to untimely balance adjustment when walking under the guidance of a fixed rhythm remains unclear. The care of medical staff during the experiment can greatly prevent such incidents from happening, but whether such incidents be avoided at home remains to be investigated. Therefore, further studies on the effectiveness and safety report of RAS for the treatment of PD in home rehabilitation are necessary.

As an effective intervention measure, RAS can be applied to home rehabilitation under the supervision and guidance of professional medical staff. The intervention method does not involve complex machinery and equipment and is easy to operate and apply. The patient only needs to wear a simple RAS device to complete it. The patients also need to go to the hospital or rehabilitation department regularly to assess their condition and adjust the frequency of stimulation to suit the patient's next treatment. RAS can save patient's time and energy and reduce the financial burden of the family.

## Study Limitations

Our findings are based on articles written in English and Chinese, and articles in other languages were not included, which may have implications for our research. In addition, possible bias may occur as some studies based the treatments on more than one cue (for example: RAS + visual stimulation) while other studies only used RAS.

## Conclusions

In summary, this study suggests the application of RAS in conventional rehabilitation approaches to enhance motor performance and quality of life in patients with PD. Future studies should use a large sample size and rigorous designs to draw strong conclusions about the advantages of RAS for the treatment of PD and promote it to family and community rehabilitation.

## Data Availability Statement

The original contributions presented in the study are included in the article/supplementary material, further inquiries can be directed to the corresponding author/s.

## Author Contributions

LW, J-lP, J-bO-Y, and LQ: concept/idea/research design. LW, J-lP, J-bO-Y, and LG: writing. LW, J-lP, and LQ: data collection. LW: data analysis. LW, J-lP, J-bO-Y, LG, SZ, H-YW, G-CZ, and LQ: consultation (including review of manuscript before submitting). All authors have read and approved the final manuscript.

## Conflict of Interest

The authors declare that the research was conducted in the absence of any commercial or financial relationships that could be construed as a potential conflict of interest.

## Publisher's Note

All claims expressed in this article are solely those of the authors and do not necessarily represent those of their affiliated organizations, or those of the publisher, the editors and the reviewers. Any product that may be evaluated in this article, or claim that may be made by its manufacturer, is not guaranteed or endorsed by the publisher.
